# Valvular regurgitation and surgery associated with fenfluramine use: an analysis of 5743 individuals

**DOI:** 10.1186/1741-7015-6-34

**Published:** 2008-11-06

**Authors:** Charles F Dahl, Marvin R Allen, Paul M Urie, Paul N Hopkins

**Affiliations:** 1 Central Utah Clinic, Department of Cardiology, Provo, Utah, USA; 2 Utah Valley Regional Medical Center, Provo, Utah, USA; 3 Cardiovascular Genetics Research Clinic, Department of Internal Medicine, University of Utah School of Medicine, Salt Lake City, Utah, USA

## Abstract

**Background:**

Use of fenfluramines for weight loss has been associated with the development of characteristic plaques on cardiac valves causing regurgitation. However, previously published studies of exposure to fenfluramines have been limited by relatively small sample size, short duration of follow-up, and the lack of any estimate of the frequency of subsequent valvular surgery. We performed an observational study of 5743 users of fenfluramines examined by echocardiography between July 1997 and February 2004 in a single large cardiology clinic.

**Results:**

The prevalence of at least mild aortic regurgitation (AR) or moderate mitral regurgitation (MR) was 19.6% in women and 11.8% in men (*p *< 0.0001 for gender difference). Duration of use was strongly predictive of mild or greater AR (*p *< 0.0001 for trend), MR (*p *= 0.002), and tricuspid regurgitation (TR) (*p *< 0.0001), as was earlier scan date (*p *< 0.0001 for those scanned prior to 1 January 2000 versus later). Increasing age was also independently associated with increased risk of AR and MR (both *p *< 0.0001). With mean follow-up of 30.3 months, AR worsened in 15.2%, remained the same in 63.1%, and improved in 21.7%. Corresponding values for MR were 24.8%, 47.4% and 27.9%. Pulmonary hypertension was strongly associated with MR but not AR. Valve surgery was performed on 38 patients (0.66% of 5743), 25 (0.44%) with clear evidence of fenfluramine-related etiology.

**Conclusion:**

Regurgitant valvulopathy was common in individuals exposed to fenfluramines, more frequent in females, and associated with duration of use in all valves assessed. Valve surgery was performed as frequently for aortic as mitral valves and some tricuspid valve surgeries were also performed. The incidence of surgery appeared to be substantially increased compared with limited general population data.

## Background

An association between valvular heart disease and the anorexic drugs fenfluramine and dexfenfluramine has been reported since the summer of 1997 [1,2] and is now widely accepted [3-6]. Nevertheless, the natural history of valvular regurgitation in former users of fenfluramines has yet to be determined. Furthermore, relatively small sample sizes have limited important subgroup analyses including comparisons between men versus women and different age categories. In addition, controversy remains regarding the effect of duration of exposure for mitral regurgitation (MR) and tricuspid regurgitation (TR), while for aortic regurgitation (AR) this relationship appears strong [6-9]. Potential progression or regression of valvular regurgitation in these patients has been reported in only a limited number of patients with relatively short (usually one year) follow-up [10-14]. Importantly, the incidence of valve surgery has not been previously reported for any well-defined cohort of individuals who have used these drugs. Recently, fenfluramine-induced valve disease requiring surgery was identified seven years after discontinuing the medication, suggesting that fenfluramine-related valvular heart disease may continue to confront clinicians [15]. Recent evidence seems to confirm the suspicion that excessive stimulation of the serotonin or 5-hydroxytryptamine (5-HT) 2B receptor results in valvular damage and regurgitation [16-18].

From July 1997 to February 2004, we collected information on a large proportion of all users of fenfluramines in the second most populous county in Utah. Preliminary results in a smaller number of these individuals were reported previously [14,19]. We report herein our observations concerning valvular regurgitation on all 5473 persons seen in our echocardiographic laboratory during this period. For the first time, we show a significant relationship between duration of fenfluramine use and MR as well as TR, and provide an estimate of the incidence of valve surgery in a large cohort exposed to fenfluramines.

## Methods

From July of 1997 through February 2004, our laboratory (at Utah Valley Regional Medical Center and at the Central Utah Clinic in Provo, Utah) was the only major center for echocardiography in Utah County, a county with a population of approximately 365,500 located just south of Salt Lake City, UT. During this period we estimate that approximately half of all the prior users of fenfluramines in Utah County were referred by their physicians to our clinic for echocardiography [20-22].

All patients undergoing transthoracic or transesophageal echocardiographic examination in our laboratory were interviewed prior to their study regarding prior use of fenfluramine and/or dexfenfluramine, including total duration of use. Demographic information including age, gender, and self-reported height and weight was also collected. Other co-morbidities such as hypertension, diabetes, or history of coronary artery disease were not consistently obtained. Reported symptoms (including shortness of breath, edema, chest pain, or dizziness) were also recorded, but not in a sufficiently rigorous, systematic manner to allow reliable correlation of change in clinical symptoms with change in echocardiographic measures. Nevertheless, no associations between these reported symptoms and degree of AR, MR, or TR were seen at baseline in the population as a whole.

In our laboratories, all echocardiograms were performed using an Acuson Sequoia C515 ultrasound machine with a 3V2C transducer. The Nyquist limit was set to a default of 0.70 m/s and the color gain was left on the 50 db preset. These defaults were used on all adult patients coming to our laboratories without further adjustment by the technicians. Echocardiograms were read using color Doppler to quantify regurgitation. Presence of trace, mild, moderate, or severe AR, MR, and TR was judged by the same semi-quantitative criteria and methods as outlined by Singh et al. [23], an approach shared by virtually all of the echocardiographic reports on fenfluramine effects to date [6]. While more quantitative scoring methods (such as effective regurgitant orifice area and regurgitant volumes) may have yielded more robust analyses, particularly for change in AR and MR, such analyses were not possible retrospectively. We have also utilized the common designation of clinically significant regurgitation referred to as 'FDA-positive' (mild or greater AR or moderate or greater MR) based on the original report from the United States Food and Drug Administration [2].

Between February 1999 and May 2000, the cardiologists reading the echocardiograms were blinded as to the patient's use of fenfluramine and/or dexfenfluramine (that is, the use or non-use of these compounds was not available to the reader on all patients coming to the laboratory until the reading was completed and recorded). During this blinded period the observed prevalence of moderate or greater MR (6.10% in 607 first-time echocardiograms) was not different than the unblinded period (5.20%). The prevalence of mild or greater AR was modestly but significantly greater during this blinded period (19.4% for blinded period versus 14.4% during the unblinded period, *p *= 0.0009), suggesting if anything, more conservative readings during the unblinded period. Importantly, the trend of increasing prevalence of AR associated with greater duration of use of fenfluramines was virtually identical for blinded and unblinded readings (both with *p *< 0.0001 for trend, data not shown). Therefore, all analyses were performed on the combined group.

Pulmonary artery systolic pressure (PASP) was calculated using the modified Bernoulli equation (PASP = 4*V*^2 ^+ RAP, where *V *= peak systolic velocity of the TR jet, RAP = right atrial pressure, and right ventricle systolic pressure assumed to equal PASP as none had pulmonic stenosis). Velocity of the TR jet was recorded by continuous wave Doppler and RAP was assumed to be 5 mm Hg[24,25] Only those considered to have pulmonary hypertension (PASP of 36 mm Hg or more, corresponding to a TR jet of 2.8 m/s or greater) had PASP recorded in the database.

Surgical information was obtained from patient records. In addition, inquiries were made of the cardiologist caring for each particular patient concerning the occurrence of valve surgery. We identified a total of 38 patients who underwent valvular replacement or repair surgery. Pathology slides on explanted valves were available and examined in 27 patients (71.1%) and 38 of 53 valves (71.7%).

Ethical approval was not required for this study, as analyses were performed using data previously collected during the course of usual clinical care and de-identified, and falls under a legal exemption for the requirement of review and approval by an Institutional Review Board (United States Code of Federal Regulations, Title 45, Part 46, section 46.101, ).

### Statistical methods

The first echocardiogram obtained was considered the index echocardiogram for each individual and was used to establish the baseline prevalence of regurgitation. In evaluating regression or progression of regurgitation, the first and last echocardiograms were used for those with two or more studies. All analyses were performed using SAS version 9.1 (SAS Institute, Inc., Cary, North Carolina). Statistical methods included chi-squared analysis, the Cochran-Armitage test for trends in univariate analyses (as implemented in PROC FREQ), Student's *t*-test (PROC TTEST), multiple logistic regression for multivariable analysis (PROC LOGISTIC), and analysis of covariance (PROC GLM). Cox regression (proportional hazards regression) was performed using PROC PHREG. The surgery-free survival time was calculated as time to surgery after the initial echocardiographic scan for those with fenfluramine-related valve surgery or time between the first scan and the end of February 2004 for those without such surgery. Surgery-free survival analysis was performed with PROC LIFETEST using the same definition for the survival time variable.

## Results

7457 echocardiograms were obtained in 5743 individuals (4825 women and 918 men). At the first encounter, the prevalence of mild or greater AR or moderate or greater MR (FDA-positive echocardiogram) in these 5743 individuals was 18.4%; gender-specific prevalence was 19.6% in women and 11.8% in men (*p *< 0.0001 for the difference between genders). The distribution of valvular lesions by age and gender is shown in Tables 1 and 2.

**Table 1:** Distribution of valvular regurgitation at initial echocardiogram in 4825 women exposed to fenfluramine and/or dexfenfluramine

	Ages							
	<20 (*n *= 25)	20–29 (*n *= 386)	30–39 (*n *= 900)	40–49 (*n *= 1442)	50–59 (*n *= 1279)	60–69 (*n *= 610)	70+ (*n *= 183)	All ages (*n *= 4825)
Aortic regurgitation								
none (%)	96.0	85.8	75.4	65.4	59.5	59.8	53.6	66.3
trace (%)	4.0	10.1	14.7	17.6	19.9	20.7	29.5	17.8
mild (%)	0	3.1	7.8	11.2	13.4	12.8	11.5	10.7
moderate (%)	0	1.0	2.0	5.1	6.6	5.7	4.9	4.7
severe (%)	0	0	0.11	0.62	0.55	0.98	0.55	0.50
Mitral regurgitation								
none (%)	64.0	42.2	36.4	29.8	24.9	22.6	18.6	29.6
trace (%)	32.0	50.8	50.7	54.4	54.3	54.1	48.6	53.0
mild (%)	4.0	4.9	10.3	11.2	13.9	13.6	13.1	11.6
moderate (%)	0	1.8	2.2	4.2	5.5	8.7	15.3	4.9
severe (%)	0	0.26	0.33	0.49	1.5	0.98	4.4	0.91
Tricuspid regurgitation								
none (%)	56.0	22.8	24.4	22.8	21.1	18.4	15.3	22.0
trace (%)	28.0	63.5	60.3	62.1	60.0	62.1	60.1	61.1
mild (%)	16.0	11.9	12.6	12.1	12.9	11.5	12.6	12.3
moderate (%)	0	1.8	2.6	2.5	5.2	6.6	10.4	4.0
severe (%)	0	0	0.11	0.55	0.70	1.5	1.6	0.62

**Table 2:** Distribution of valvular regurgitation at initial echocardiogram in 918 men exposed to fenfluramine and/or dexfenfluramine

	Ages							
	<20 (*n *= 12)	20–29 (*n *= 31)	30–39 (*n *= 125)	40–49 (*n *= 249)	50–59 (*n *= 281)	60–69 (*n *= 166)	70+ (*n *= 54)	All ages (*n *= 918)
Aortic regurgitation								
None (%)	91.7	90.3	86.4	79.5	73.7	62.0	55.6	74.6
Trace (%)	8.3	9.7	10.4	13.6	12.8	24.1	27.8	15.5
Mild (%)	0	0	2.4	4.8	12.1	8.4	13.0	7.6
moderate (%)	0	0	0.80	2.0	1.4	4.2	3.7	2.1
severe (%)	0	0	0	0	0	1.2	0	0.22
Mitral regurgitation								
none (%)	58.3	58.1	48.0	44.2	35.9	34.9	44.4	41.2
trace (%)	33.3	35.5	48.0	48.6	54.4	47.6	46.3	49.4
Mild (%)	8.3	3.2	4.0	7.2	7.5	9.6	5.6	7.1
moderate (%)	0	0	0	0	1.1	6.0	3.7	1.6
severe (%)	0	3.2	0	0	1.1	1.8	0	0.76
Tricuspid regurgitation								
none (%)	41.7	38.7	37.6	34.1	31.7	26.5	31.5	32.5
trace (%)	33.3	51.6	60.0	56.6	62.6	59.6	55.6	59.0
Mild (%)	16.7	9.7	2.4	7.2	4.3	9.0	9.3	6.3
moderate (%)	0	0	0	2.0	0.71	4.2	3.7	1.7
severe (%)	8.3	0	0	0	0.71	0.60	0	0.44

**Figure 1:**
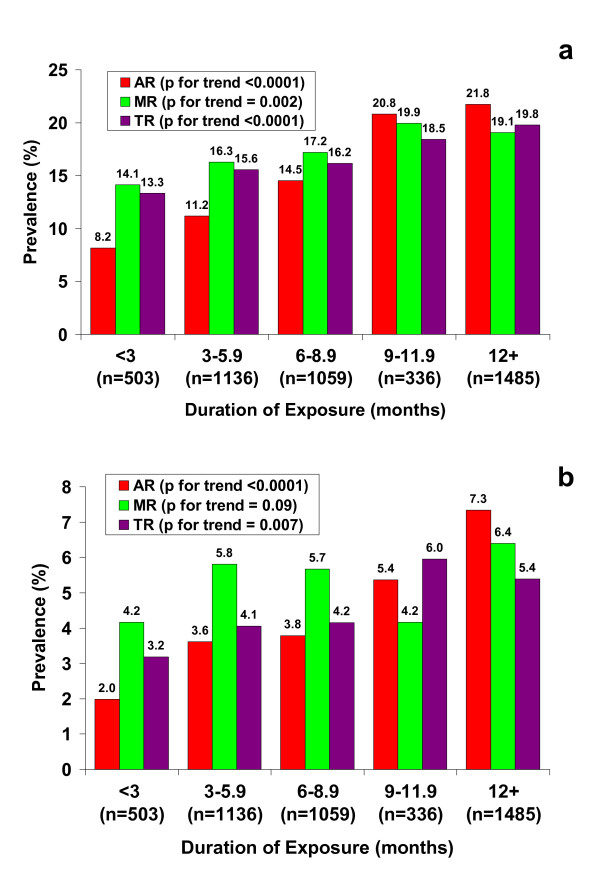
**Relationship between duration of exposure and valvular regurgitation**. a) Prevalence of mild or greater aortic regurgitation (AR), mitral regurgitation (MR), and tricuspid regurgitation (MR) as a function of duration of fenfluramine use. *P*-values are for one-sided trend test. b) Prevalence of moderate or greater aortic regurgitation (AR), mitral regurgitation (MR), and tricuspid regurgitation (MR) as a function of duration of fenfluramine use.

We first explored factors that were associated with valvular regurgitation at the baseline echocardiogram. As noted above, FDA-positive valvular regurgitation was more frequent in women compared with men. Duration of use of fenfluramines was obtained in 4519 individuals. As shown in Figure 1, there was a striking trend of increasing prevalence of mild or greater AR associated with greater duration of fenfluramine use (*p *< 0.0001 for trend, one-sided). Mild or greater MR and TR were also significantly associated with duration of use (Figure 1a). Because only moderate or greater MR and TR are generally considered clinically important, we also show trends for moderate or greater AR, MR, and TR in Figure 1b. The association of duration of use remained highly significant for moderate or greater AR (*p *< 0.0001) but the association with moderate or greater MR did not reach statistical significance (*p *= 0.09, one-sided) while the trend for moderate or greater TR remained statistically significant (*p *= 0.007, one-sided). Mild or greater AR and MR were more likely present for scans performed prior to 1 January 2000 (20.5% and 24.5% respectively) than scans performed on or after this date (prevalence for both AR and MR was 12.1% with differences between earlier and later prevalence both significant at *p *< 0.0001).

In multiple logistic regression utilizing baseline scans, the presence of mild or greater AR was significantly associated with age (odds ratio (OR) = 1.30 per decade, 95% CI 1.21–1.39, *p *< 0.0001), gender (OR = 1.77 for female gender, 95% CI 1.37–2.29, *p *< 0.0001), duration of use of fenfluramines (OR = 1.32 for three-month increment as shown in Figure 1, 95% CI 1.24–1.40, *p *< 0.0001), and time elapsed between the date fenfluramine was taken off the market (15 September 1997) and the date that the scan was performed (OR = 0.94 per year, 95% CI 0.90–0.98, *p *= 0.006).

Mild or greater MR at baseline was significantly associated with age (OR = 1.35 per decade, 95% CI 1.27–1.44, *p *< 0.0001), gender (OR = 2.26 for female gender, 95% CI 1.74–2.92, *p *< 0.0001), duration of use (OR = 1.089 per month, 95% CI 1.031–1.151, *p *= 0.002), and time elapsed (OR = 0.89 per year, 95% CI 0.86–0.93, *p *< 0.0001). Moderate or greater MR at the baseline scan was associated only with age (OR = 1.42 per decade, 95% CI 1.35–1.50, *p *< 0.0001), and gender (OR = 3.08 for female gender, 95% CI 1.98–4.80, *p *< 0.0001).

There were 1020 individuals who had two or more echocardiograms. The average time between the first and last study in these individuals was 30.3 months. For individual valves, AR increased in 15.4%, stayed the same in 63.1%, and improved in 21.5%. For MR, 24.8% worsened, 47.6% showed no change, and 27.6% improved. For TR, corresponding percentages were 27.6%, 48.1%, and 24.2% respectively. Examining change in regurgitation as a continuous variable in analysis of covariance (with none, trace, mild, moderate, and severe assigned values of 0, 1, 2, 3 and 4 respectively), of the available predictors only greater age was significantly associated with worsening regurgitation (*p *= 0.05 for change in AR, *p *= 0.04 for MR, and *p *= 0.0006 for TR) considering valves individually and adjusting for baseline regurgitation. Greater initial regurgitation was associated with greater improvement in the second scan for all valves (*p *< 0.0001 for all). Greater time elapsed between the baseline scan and removal from the market also predicted greater improvement for all valves (*p *= 0.0009 for AR, *p *< 0.0001 for MR, and *p *= 0.0008 for TR). Time between scans was not significantly associated with change of regurgitation in any of these models.

Final degree of regurgitation as a function of initial regurgitation is given for aortic valve in Table 3 and for mitral valve in Table 4. Overall, of the 620 who were not FDA-positive at baseline, 63 (10.2%) became FDA-positive, while of the 400 who were FDA-positive at baseline, 131 (32.8%) became FDA-negative. The presence of mild or greater AR at the final visit remained significantly associated with duration of fenfluramine use (*p *= 0.009, one-sided test for trend), while the association with moderate or greater MR remained non-significant.

**Table 3:** Number (percent of total) of persons with aortic regurgitation at baseline and final echocardiograms

Baseline	Final					
	None	Trace	Mild	Moderate	Severe	Totals
None	425	74	11	7	0	517
	(41.67)	(7.25)	(1.08)	(0.69)	0	(50.69)
Trace	56	90	13	8	0	167
	(5.49)	(8.82)	(1.27)	(0.78)	(0)	(16.37)
Mild	19	66	79	36	3	203
	(1.86)	(6.47)	(7.75)	(3.53)	(0.29)	(19.90)
Moderate	6	21	41	45	5	118
	(0.59)	(2.06)	(4.02)	(4.41)	(0.49)	(11.57)
Severe	0	0	2	8	5	15
	(0)	(0)	(0.20)	(0.78)	(0.49)	(1.47)
Totals	506	251	146	104	13	1020
	(49.61)	(24.61)	(14.31)	(10.20)	(1.27)	(100)

**Table 4:** Number (percent of total) of persons with mitral regurgitation at baseline and final echocardiograms

Baseline	Final					
	None	Trace	Mild	Moderate	Severe	Totals
None	84	155	16	4	1	260
	(8.24)	(15.20)	(1.57)	(0.39)	(0.10)	(25.49)
Trace	90	300	31	16	2	439
	(8.82)	(29.41)	(3.04)	(1.57)	(0.20)	(43.04)
Mild	24	95	57	22	2	200
	(2.35)	(9.31)	(5.59)	(2.16)	(0.20)	(19.61)
Moderate	7	32	16	40	4	99
	(0.69)	(3.14)	(1.57)	(3.92)	(0.39)	(9.71)
Severe	0	0	6	12	4	22
	(0)	(0)	(0.59)	(1.18)	(0.39)	(2.16)
Totals	205	582	126	94	13	1020
	(20.10)	(57.06)	(12.35)	(9.22)	(1.27)	(100)

We assessed the association of pulmonary hypertension with available covariates at baseline. As PASP was recorded only for those considered to have pulmonary hypertension (PASP 36 mm Hg or higher), we evaluated correlates of presence or absence of pulmonary hypertension. There were 150 subjects (2.6% of all patients) judged to have pulmonary hypertension at baseline. Among these patients, mean PASP was 47.3, SD 8.3, range 36 to 85 mm Hg. Associations with AR were generally inconsistent. Prevalence of pulmonary hypertension among those having no AR was 2.03% and there was no significant trend for increasing AR grades (3.49%, 3.76%, 5.74%, and 3.85% respectively). In contrast, for increasing MR there was a very strong gradient of association (*p *for trend < 0.0001) as shown in Table 5, with odds ratios from multiple logistic regressions being significant for all grades of MR (as compared with no MR). Age was also strongly associated with presence of pulmonary hypertension (*p *< 0.0001, OR 1.039 per year of increasing age, 95% CI 1.025–1.054) while gender was of borderline significance (*p *= 0.09, OR 1.6 for female gender, 95% CI 0.92–2.9), as was body mass index (BMI) (OR 1.004 per BMI unit increase, 95% CI 1.000–1.008, *p *= 0.08). Among the 4519 patients with duration of exposure data available, we found a significant association between pulmonary hypertension and duration of exposure (OR 1.030 per month of exposure, 95% CI 1.008–1.051, *p *= 0.006), which was independent of MR grade. Alternatively, the OR for exposure of six months or longer (as compared with under three months) was 2.5 (95% CI 1.13–5.4, *p *= 0.0007) while for exposure of three to under six months it was non-significant (OR 1.13, 95% CI 0.5–2.7, *p *= 0.3).

**Table 5:** Association between baseline mitral regurgitation and presence of pulmonary hypertension (PHTN) (defined as pulmonary systolic pressure 36 mm Hg or higher)

	Mitral regurgitation at baseline				
	
	None	Trace	Mild	Moderate	Severe
Total	1804	3011	624	253	51
PHTN present (n)	12	59	36	32	11
PHTN absent (n)	1792	2952	588	221	40
PHTN (%)	0.67	1.96	5.77	12.65	21.57
OR for PHTN	1	2.7 (1.4–5.0)	7.6 (3.9–15)	15 (7.4–30)	28 (12–69)

Odds ratio (OR) for PHTN was derived by multiple logistic regression with (95% CI), adjusting for age and gender. Shown are both numbers (n) and percent (%) with PHTN.

Valve surgeries were performed on 53 valves (48 replacements and five repairs) in 38 individuals. Surgical reports were available for all. There were 23 aortic valve surgeries (no repairs), 27 mitral valve surgeries (five repairs) and three tricuspid valve surgeries (one repair). The incidence of surgery overall was 0.66% (38 of 5743 individuals). In women the incidence was 0.64% (31 of 4825) and in men it was 0.76% (seven of 917). The mean age at the baseline echocardiogram of those with subsequent valve surgery was 55.6 years (range 40 to 75).

**Figure 2:**
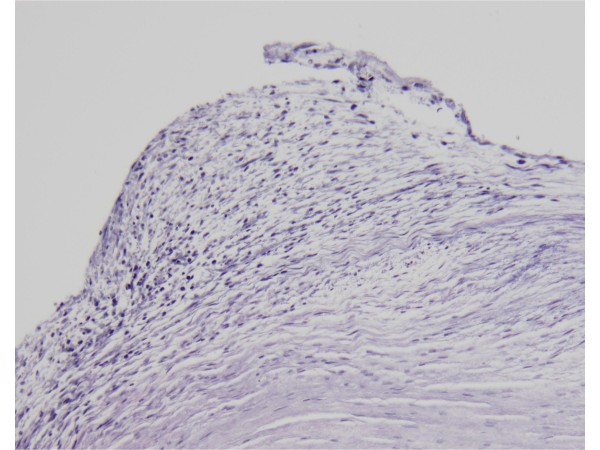
Example of a microscopic section of EVG-stained mitral valve showing histopathology typical of fenfluramine-related changes.

Pathology specimens were available for review for 38 valves (27 individuals) (see Tables 6 and 7). To be considered positive for fenfluramine-related pathology a valve and/or chordae tendineae had to have features of valve thickening by gross examination and fibrous plaques 'stuck-on' an intact valve architecture by microscopic examination. Hematoxylin and eosin stained sections were available to study in each of the 38 valves examined. In most of the cases, Masson trichrome stain and Verhoeff's elastic van Gieson (EVG) stain were also available for review (see Figure 2). The valve sections on the slides were not oriented, but valve architecture was adequately evaluated. Other valve pathologic processes were noted if present. As described previously [26], plaques made up of a myxoid extracellular matrix with myofibroblasts were seen on valves in the affected patients. Calcification was absent. Pathology was consistent with fenfluramine-related damage in 25 valves (11 aortic, 13 mitral and one tricuspid) and negative in 13 valves (eight aortic and five mitral). Four valves demonstrated a mixture of both fenfluramine-related pathology and other pathology. Other valve pathologic processes included but were not limited to post-inflammatory scarring and myxomatous degeneration. Two individuals demonstrated fenfluramine-related pathology on only one of the two valves examined pathologically.

**Table 6:** Apparent etiology of valvulopathy in persons undergoing valvular surgery by gender

	Fenfluramine related (*n *= 25)		Not fenfluramine related (*n *= 13)		
			
	Pathology	Clinical	Pathology	Clinical	Total
Women	16	7	6	2	31
Men	2	0	3	2	7
Totals	18	7	9	4	38

Pathology refers to microscopic examination of valve tissue obtained at surgery. Clinical refers to gross appearance of valve at surgery or clinical evaluation (echocardiographic appearance, findings of rheumatic disease, aortic stenosis, and other recognized valve disease other than fenfluramine-related).

**Table 7:** Apparent etiology of valvulopathy in persons undergoing valvular surgery by valve

	Fenfluramine related (*n *= 36 valves)		Not fenfluramine related (*n *= 17 valves)		
			
	Pathology	Clinical	Pathology	Clinical	Total
Aortic	11	3	8	1	23
Mitral	13	6	5	3	27
Tricuspid	1	2	0	0	3
Total	25	11	13	4	53

In 15 valves (11 individuals) pathology specimens were not available for review. These were adjudicated to be probably positive or probably negative for fenfluramine-related etiology of valvular disease based on clinical, echocardiographic, and gross surgical findings. Eleven valves (seven patients) were judged to have a probable fenfluramine-related etiology while four were not.

**Figure 3:**
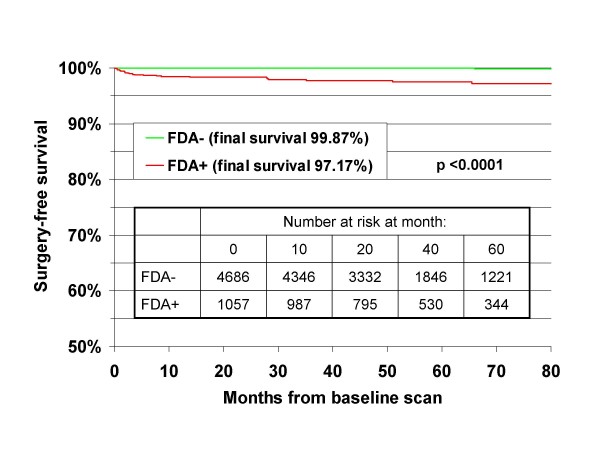
**Surgery-free survival (restricted to the 25 surgeries clinically or pathologically judged as due to fenfluramine exposure)**. Comparison is made between those with baseline mild or greater aortic regurgitation or moderate or greater mitral regurgitation (FDA+) versus those with less or no regurgitation (FDA-). 23 of the 25 surgeries occurred in those who were FDA+ at baseline. The difference in surgery-free survival between the groups was highly significant (*p *< 0.0001 by log-rank test).

If one considers rates of surgery only in the 25 individuals with definite or probable fenfluramine-related pathology, the incidence in the overall cohort was 0.44%. In women, the incidence was 0.48% and in men the incidence was 0.22% (*p *= 0.4 for gender difference) during the 6.5 years of follow-up. Mean age in this group was 54.3 years (range 40 to 67). In Cox regression analyses, neither age at baseline, gender, nor duration of exposure was significantly related to surgery. Degree of regurgitation at baseline was highly predictive of subsequent surgery. Compared with lower grades or no regurgitation there were increased risks of valve surgery for persons with mild (OR = 4.6, 95% CI 1.5–14.6, *p *= 0.009), moderate (OR = 7.9, 95% CI 2.6–23.8, *p *= 0.002) and severe (OR = 52.8, 95% CI 14.5–193, *p *< 0.0001) AR; with moderate (OR = 9.6, 95% CI 3.5–26.3, *p *< 0.0001) and severe (OR = 40.7, 95% CI 14.2–117, *p *< 0.0001) MR; and with mild or greater (OR = 3.1, 95% CI 1.3–7.8, *p *= 0.015) TR. Mild or greater AR or moderate or greater MR (FDA-positive regurgitation) was associated with an odds ratio of 48.7 (95% CI 11.5–207, *p *< 0.0001) for valve surgery. Surgery-free survival curves comparing those with and without FDA-positive regurgitation at baseline are shown in Figure 3. The final (80-month) surgery-free survival in the 4686 persons initially without FDA-positive regurgitation was 99.87% while for the 1057 with FDA-positive regurgitation at baseline it was 97.17%. These differences in the surgery-free survival were highly significant (*p *< 0.0001 by log-rank test).

## Discussion

This is the largest study to examine duration of exposure to fenfluramines and the first to estimate the incidence of valvular surgery among prior users of fenfluramines. In addition, this is the first study to identify clearly greater risk associated with fenfluramine use in women. We found clear evidence for a strong, graded association between duration of exposure to fenfluramines and prevalence of AR and for mild or greater MR and TR. Associations with TR have apparently not been previously examined. For MR, prior studies have generally used moderate or greater regurgitation as the cut point, coinciding with clinical importance but resulting in lower power. As in prior studies, mild or greater aortic regurgitation was the most common finding in these individuals at baseline. Interestingly, severe AR, MR, and TR had similar prevalence. It is also noteworthy that both aortic and mitral valve surgery had a similar incidence.

Both regression and progression of disease was seen in these individuals. It has been reported that regression occurs in about one-quarter to one-third of individuals [12-14,27]. Our data are consistent with these observations. Improvement in regurgitation appeared to be more common than worsening. Some of this apparent improvement is likely due to physiologic regression to the mean; that is, the result of random differences in degree of regurgitation expected from one scan to another. Importantly, most regurgitation remained unchanged over the period of 30 months in our study. Furthermore, some degree of latency in the emergence of regurgitation is suggested in that 17% of patients without either mild or greater AR, moderate or greater MR, or moderate or greater TR on first echocardiogram demonstrated such findings on second examination. Considering only FDA criteria (mild or greater AR or moderate or greater MR), 10.2% who were not FDA-positive initially became FDA-positive on follow-up. Progression of regurgitation, despite removal of fenfluramine, has been documented in a heart transplanted from a former fenfluramine user [28], and valvulopathy requiring surgery seven years after stopping the drug has been reported [15]. This prolonged effect is currently thought to be due to direct stimulation of 5-HT2B receptors by norfenfluramine, a metabolite of fenfluramine which binds avidly and may persist in tissues, resulting in promotion of mitogenesis of valvular fibroblasts [16,29-33].

TR in fenfluramine users has received little attention. While we report the prevalence of moderate or severe TR, we did not include TR when defining which individuals were considered FDA-positive (defined as mild or greater AR or moderate or greater MR). However, the prevalence of moderate or greater TR was increased compared with published prevalence in the general population [23] and was similar to prevalence of MR in our population. Importantly, this is the first study to demonstrate increasing prevalence of TR associated with greater duration of fenfluramine use. In addition, characteristic fenfluramine-related pathology was documented at surgery for the tricuspid valve in one individual.

We believe the strong correlation we observed between MR grade and the presence of pulmonary hypertension lends credence to the physiological and clinical significance of our assessment of MR. The association between MR and pulmonary hypertension in the setting of prior fenfluramine users has not been previously reported to our knowledge (though such a relationship might have been anticipated based on physiological considerations). While use of fenfluramines is a well-known cause of severe primary pulmonary hypertension [34,35], duration of exposure to fenfluramines as a correlate to generally more modest pulmonary hypertension in otherwise unselected fenfluramine users has not been previously reported. Importantly, this relationship was seen even after adjustment for MR grade in multiple logistic regressions. Graded fibroproliferative changes of pulmonary arteries would be consistent with current understanding of pathophysiology, including involvement of the 5-HT2B receptor as well as predisposing conditions such as hypoxia and genetic factors, which seem to be associated with increased receptor expression prior to exposure [36-43]. A strong association between age and PASP as well as a more modest association with BMI in fenfluramine users has been previously reported and is consistent with our observations [25]. Due to limited data on potential interventions and reasons for not having a second examination, we did not examine correlates of change of pulmonary hypertension status. However, the association between MR grade and pulmonary hypertension was similar at the final scan in those with more than one scan as at baseline (data not shown).

We recognize the need to consider potential sources of bias in our study. Types of bias to consider include: how subjects were ascertained or selected (so-called selection bias or referral bias could operate to increase prevalence if individuals who were more ill or symptomatic chose to see their primary care physicians more often, or were referred selectively to our clinic by their physicians); how the scan was performed by the sonographer (referred to as acquisition bias, this could occur if there was inappropriate, systematic, or selective adjustment of gains upward to exaggerate regurgitant jets in those exposed to fenfluramines); and how the scan was read by the cardiologist (interpretation bias).

**Figure 4:**
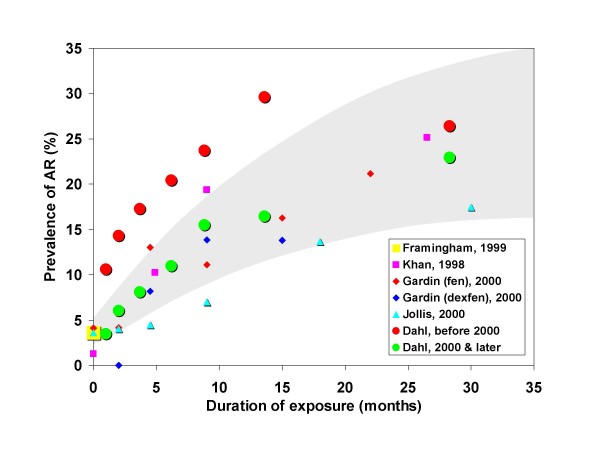
**Prevalence of mild or greater aortic regurgitation (AR) as a function of duration of exposure to fenfluramines**. The shaded area is the 95% confidence interval based on pooled prevalence rates from studies by Khan et al [7], Gardin et al [8], and Jollis et al [9]. These were 'controlled' studies that included an unexposed group. Note the generally higher rates reported in the earlier Khan report. For comparison purposes, an estimate of the expected, unexposed prevalence (3.6%) was derived, using the direct method, from the Framingham study (Singh et al [23]) based on the age and gender distribution of our own study. Our own cohort was divided by proximity of the scan to withdrawal of fenfluramine from the market (on 15 September 1997) with *N *= 1890 scans prior to 1 January 2000 and the remaining (*N *= 3853) on or after that date.

We previously noted (see Methods) that during a blinded period when cardiologists were reading scans without knowledge of fenfluramine use, diagnosis of regurgitation was, if anything, more frequent than during the un-blinded period. Thus, interpretation bias may be safely excluded. Sonographers were experienced employees of our clinic with no incentive to over-diagnose regurgitation. Furthermore, sonographers did not adjust machine settings from defaults. For acquisition bias to explain our findings, sonographers would have had to systematically increase gain settings for patients with longer duration of exposure in a graded fashion, and systematically skew detection for patients who were older, female, and were scanned at dates closer to the time they took fenfluramine, and this would have to have been done in only a plausible and graded percentage of scanned patients. Thus, while acquisition bias has been cited by skeptics as a source of overall higher rates (which is unlikely for reasons discussed below), it defies credulity as the source for the graded associations with external factors we report herein. Furthermore, among the 112 subjects with very limited exposure (one month or less) the prevalence of mild or greater AR (3.48%) was virtually identical to the expected value from the Framingham population [23] adjusted for age and gender by the direct method (see Figure 4). In this group with one month or less exposure, prevalence of mild or greater MR (12.2%), and moderate or greater TR (2.61%) was not significantly greater than expected from the Framingham population. Moderate or greater MR (4.35%) in this group was significantly (*p *= 0.002) more frequent than reported by the Framingham study [23] but not different than reported in some other control populations [6].

Selection bias, though the most concerning theoretical bias for our study, was an unlikely explanation of our results. As noted previously, approximately half of all persons using fenfluramines in the catchment area of our clinic (primarily Utah County) came to us for echocardiograms, probably due to aggressive screening efforts prompted by FDA recommendations [2]. While inclusion of such a large proportion of those exposed may be reassuring, the strongest test for significant bias is direct comparison with 'controlled' studies ostensibly free of bias. For comparison with our own findings we examined all studies reporting prevalence of AR at more than one exposure level and which included an unexposed group [7-9]. Note here that these studies were not case-control studies. Rather, they included an unexposed group typically referred to as a control group. In Figure 4 we show the reported prevalence of mild or greater AR by exposure duration from each of these studies together with a 95% confidence interval estimated from the pooled prevalence at each exposure level. For our own data, we have plotted results grouped by baseline scan dates before or after 1 January 2000. As is readily apparent in Figure 4, prevalence from our later scans exactly coincides with the expected prevalence from contemporaneous cohorts. Note too that our group with very low exposure (one month) had prevalence rates of mild or greater AR virtually identical to both the unexposed groups of cited studies [7-9] and to an age and gender-weighted estimate of expected prevalence from Framingham [23] (also plotted in Figure 4). Since each form of bias discussed above, if present, would be expected to spuriously increase prevalence of regurgitation, the lack of such excessive prevalence argues strongly against any substantial bias. Furthermore, the remarkable consistency of dose response among this and other reports is consistent with a biologically plausible effect. We therefore conclude that our cohort was reasonably representative of fenfluramine users in Utah and elsewhere.

The much higher prevalence of AR in our earlier scans deserves further comment. It should be noted that over 80% of those in our pre-2000 group had scans in 1997 and 1998 and thus most of these earlier scans were performed in relatively close proximity to the time of taking fenfluramines. While frequently disparaged as 'uncontrolled', data from five independent groups included in the original FDA report found highly consistent prevalence rates of mild or greater AR or moderate or greater MR that averaged 32.8% among persons concurrently taking fenfluramines [2]. Furthermore, there was a concerning trend for increasing prevalence with longer exposure even with very limited data at that time. Others have reported very high prevalence of regurgitation in those recently or currently taking fenfluramine with marked improvement to more modest levels after more prolonged follow-up off treatment [13,20,27]. These changes were particularly evident in the aortic valve. Our findings are highly consistent with these reports. Therefore, the higher rates we observed with our earlier scans (see Figure 4) should not be construed as evidence for bias.

These observations suggest the hypothesis that there may be a component of fenfluramine-related regurgitation that is reversible soon after stopping the drug. Supporting evidence comes from studies showing aortic cusps to have contractile elements, and that exposure of isolated porcine aortic valves to serotonin resulted in acute regurgitation, a phenomenon that might be expected to be rapidly reversible [44,45]. Regurgitation based on structural lesions would be expected to be less reversible and responsible for the lower, more stable, but still excessive rates reported by later 'controlled' studies and our own study. This hypothesis would harmonize virtually all observations to date on the prevalence of AR and MR in those taking fenfluramines.

Our estimate of the incidence of valvular surgery was determined by chart review of actual surgical and pathology reports. As such, we were dependent on the records available and could have missed an unknown number of patients who may have had surgery or follow-up elsewhere. Therefore, our estimate is conservative and likely lower than the true incidence of valve surgery. While the incidence of mitral surgery has been reported to be greater than aortic surgery in some case series [26,46], aortic and mitral valve surgeries occurred at similar rates in our cases.

Clearly, rates of valve surgery in unexposed persons of a similar age distribution would be too low for any single center to provide stable estimates. We are therefore obliged to use very limited data available from large, population-based studies as a relatively informal comparison. The only population-based data available appear to be from the Medical Device Implant Supplement to the 1988 National Health Interview Survey (122,310 individuals surveyed) [47]. The prevalence of prosthetic heart valve recipients was 2.0 per 1000 in the 45 to 64 year age category. This rose to 4.2 in the 65 to 74 year age group and was only 0.2 per 1000 in those under 45 years old. Only 10% of the surgeries had occurred in the year prior to the survey, giving an expected rate of valve surgery of 0.02% per year in those 45 to 64 years of age. In addition, 52% of the surgeries were for conditions that we could rule out in our surgical cases, including rheumatic heart disease (23%), congenital anomalies (15%), complications of myocardial infarct (12%), valve calcification (6%), and endocarditis (2%). We would therefore expect to see in 6.5 years of follow-up of our population, (0.02%)(6.5 years)(48%)(5743) = 3.58 cases. Considering there were 25 surgical cases in our series with pathologically demonstrated or clinically suspected fenfluramine-related pathology (that is, excluding the above problems), the apparent risk for valve surgery was increased approximately seven-fold (*p *< 0.0001 by Poisson). That this is probably a conservative estimate is underscored by findings from the only prospective, population-based study of fenfluramine use which reported a 17- to 34-fold excess of clinically apparent (presumably severe), idiopathic valvular disease in persons using fenfluramines for four months or longer compared with those using these drugs for a shorter period or not at all [6,48].

## Conclusion

Our findings bring considerable harmony and closure to the issue of fenfluramine-induced valvulopathy. A strong association with duration of exposure was seen not just for AR (as reported by others), but also for MR and TR as well. Prior negative findings for a graded association with MR were likely due to undue focus on only moderate or greater regurgitation with resulting loss of power. Our finding of a strong effect of timing of the scan (in relation to stopping the medication) lends credibility to the high prevalence of early reports and is also in harmony with the lower, stable rates seen in later studies. We found evidence for early improvement followed by relative stabilization with only slightly more individuals showing improvement over the longer term as compared to deterioration. Pulmonary hypertension has generally not been examined closely in prior studies of fenfluramine-related valvulopathy, but our findings are consistent with an exposure-related fenfluramine effect as well as a strong, direct MR effect. Finally, valvulopathy requiring surgery, while not common in absolute terms, was substantially increased above expected rates and may continue to be seen by clinicians well into the future.

## Competing interests

CFD, PMU, and PNH have served in the past as expert witnesses for plaintiffs in fenfluramines-related litigation. It should be noted that this litigation has concluded and there are no financial considerations that could be considered competing interests. This work was not supported by any outside funding.

## Authors' contributions

CFD oversaw collection of all data, suggested several analyses, assisted in data interpretation, and helped to write and edit the manuscript. MRA contributed to editing the manuscript. CFD and MRA read the majority of the scans. PMU performed all pathological examinations of explanted valves and assisted in editing the manuscript. PNH performed all statistical analyses, suggested analyses, assisted in data interpretation, and helped to write and edit the manuscript. All authors read and approved the final manuscript.

## Pre-publication history

The pre-publication history for this paper can be accessed here:


